# Development of MRI Projection Mapping System for Breast-Conserving Surgery in the Operating Room: Preliminary Clinical Results in Invasive Breast Cancer

**DOI:** 10.1155/2020/5314120

**Published:** 2020-06-29

**Authors:** Maki Amano, Toshiaki Kitabatake, Otoichi Nakata, Yuko Ichikawa, Reiko Inaba, Kazuyuki Ito, Kanako Ogura, Yutaka Ozaki, Kuniaki Kojima, Shigeki Aoki, Ryohei Kuwatsuru

**Affiliations:** ^1^Department of Radiology, Juntendo Nerima Hospital, 3-1-10 Takanodai, Nerima-ku, Tokyo 177-8521, Japan; ^2^Department of Radiology, Juntendo University School of Medicine, Tokyo, Japan; ^3^Department of Breast Surgery, Juntendo Nerima Hospital, Tokyo, Japan; ^4^RICOH Company, Ltd., Kanagawa, Japan; ^5^Department of Pathology, Juntendo Nerima Hospital, Tokyo, Japan

## Abstract

**Aim:**

To evaluate the feasibility of a newly developed prototype MRI projection mapping (PM) system for localization of invasive breast cancer before breast-conserving surgery.

**Methods:**

This prospective study enrolled 10 women with invasive breast cancer. MRI was performed in both prone and supine positions. The tumor location was drawn on the breast skin using palpation and sonography while referring to the prone MRI (i.e., a conventional method). A maximum intensity projection image generated from the supine MRI was projected using our PM system, and the tumor location was drawn. The PM system consisted of a projector and a camera and was used to measure the shape of the breast surface using the structured light method. Breast-conserving surgery was performed based on the conventional method. We compared the tumor size and location between the PM and conventional methods or pathology.

**Results:**

There were no significant differences in the maximum diameters of invasive cancers between the PM system and the conventional method or pathology. The maximum discrepancy in tumor location between the PM and conventional method was 3–8 mm.

**Conclusions:**

This PM system may support breast-conserving surgery by showing the tumor size and location on the breast surface.

## 1. Introduction

Breast-conserving surgery has been established as a standard treatment for relatively small breast cancer [1]. In such surgery, a positive surgical margin is associated with local recurrence and poor prognosis [2, 3], while it is required to maintain the shape of the breast as much as possible. Therefore, it is important to determine the location and spread of the tumor accurately.

The location and spreading of breast cancers are assessed using mammography, sonography (US), and MRI. MRI is the best modality for depicting the spread of breast cancer, including ductal carcinoma in situ (DCIS), which spreads in three-dimensional (3D) directions [4, 5]. Maximum intensity projection (MIP) images generated from contrast-enhanced 3D MRI allow for comprehensive identification of the tumor location. Therefore, contrast-enhanced MRI is usually performed before breast-conserving surgery to estimate the spread of breast cancer accurately. Nonetheless, the surgical margins for breast-conserving surgery are determined conventionally by palpation and US while referring to MRI findings in the operating room (OR) (i.e., the conventional method). Based on the tumor location and resection line drawn on the breast skin using the conventional method, the cylindrical resection of the cancer is performed when not using hook-wire-guided localization. Because routine MRI is performed with the patient in a prone position, breast surgeons cannot use its information for tumor localization directly. Actually, the shape of the breast is quite different between the prone position required during MRI scans and in a supine position on the operating table. Therefore, the location and spread of breast cancers must change from the time of MRI scans to the OR [6, 7]. It requires labor and experience for breast surgeons to reconstruct the spread of breast cancer and to determine the optimal surgical line from MRI.

We have developed a prototype projection mapping (PM) system that consisted of a projector and a camera. This is used to measure the shape of the breast surface by the structured light method [8, 9] and can project the MIP image of a breast MRI scan taken in a supine position onto the breast skin. The aim of this study was to evaluate whether the location and spread of invasive breast cancer shown by the PM method was consistent with those identified by conventional methods or pathological findings. In this study, we focused on invasive breast cancer identified by US to observe the accuracy of this system because the US approach used in the conventional method can identify invasive tumors well, but not necessarily DCIS.

## 2. Materials and Methods

### 2.1. Patients

This prospective study was approved by the Institutional Review Board of our institution. Consequently, written informed consent was obtained from 10 female patients from October 2017 to March 2019. All of them had invasive breast cancer identified by US. Their age ranged from 36 to 70 years (mean, 55.6). The location, histology, and clinical stage of their breast cancers are shown in Table 1.

### 2.2. MRI

**Figure 1:**
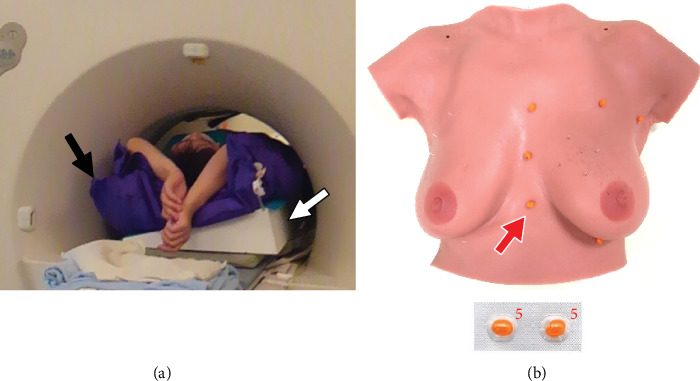
Patient positioning. (a) Contrast-enhanced breast MRI is performed with the patient in a supine position. The arms of the patient are raised using Vac-Lok™ cushions (black arrow) to reproduce their position on the operating table. A home-made tilting table with a 15° slope (white arrow) is set under the back of the patient with breast cancer in the outer breast region. (b) Markers are put on the rigid chest walls. Adalat capsules are hyperintense on contrast-enhanced fat-suppressed T1-weighted images.

MRI was performed using a 1.5 T unit (Signa Excite, GE Healthcare, Milwaukee, WI, USA). Dynamic transverse fat-suppressed T1-weighted three-dimensional (3D) fast gradient-echo images (LAVA, GE Healthcare) with the patient in a prone position were taken with a 4-channel breast coil routinely. The LAVA was sequentially acquired before and 3 phases after administration of the contrast medium. For the dynamic MRI, gadobutrol (Gadovist; Bayer, Osaka, Japan) was administered intravenously using a power injection at a dose of 0.1 mmol/kg of body weight and a flow rate of 1 mL/s, followed by flushing with 20 mL saline. The parameters of the sequence for LAVA were as follows: repetition time (TR), 6.7 ms; echo time (TE), 3.2 ms; flip angle, 10; field of view (FOV) 340 × 340 mm^2^; matrix, 384 × 256; thickness 3 mm with 1.5 mm overlap; one acquisition; and an acquisition time of 110 s. In addition, sagittal fat-suppressed high-resolution T1-weighted 3D fast gradient-echo image was acquired between the 2nd and 3rd phases of the dynamic scan in the breast involved by cancer. The imaging parameters were as follows: TR, 15.2 ms; TE, 2.2 ms; flip angle, 15; FOV, 180 × 180 mm^2^; matrix, 256 × 160; thickness 2 mm with an overlap of 1 mm; one acquisition; and an acquisition time of 110 s. One day before surgery, contrast-enhanced breast MRI with the patient in a supine position was performed with an 8-channel cardiac coil; only the breast with invasive cancer was scanned in this sequence. The patient's arms were raised using Vac-Lok™ custom-formed vacuum cushions (Toyo Medic, Tokyo, Japan) commonly used for radiation therapy to reproduce the arms' position on the operating table (Figure 1(a)).

A home-made tilting table with a 15° slope was set under the back of the patient with the cancer positioned in the outer region of the breast. Next, rigid skin markers were put on the chest wall surface, i.e., three points vertically on the sternum and the upper and lower extramammary chest walls (Figure 1(b)). We added markers to the lateral chest walls in the patients with cancer in the outer region of the breast. Adalat® 5 mg capsules (Bayer, Osaka, Japan) were used as skin markers because they were bright on contrast-enhanced fat-suppressed T1-weighted images. The scan parameters of transverse were as follows: TR, 4.48 ms; TE, 2.14 ms; flip angle, 15°; FOV, 280 × 280 mm^2^; matrix size, 512 × 512; and slice thickness, 2 mm; and scan time, 2 min 44 s. This contrast-enhanced breast MRI was performed at 20 s delay after an intravenous injection of gadolinium-based contrast medium (0.1 mmol/kg of gadobutrol). MIP images were generated at 5° intervals from 0 to 45° and transformed to the PM system. The location of each Adalat® capsule on the chest wall skin was marked using a pen before removing it.

### 2.3. PM System

**Figure 2:**
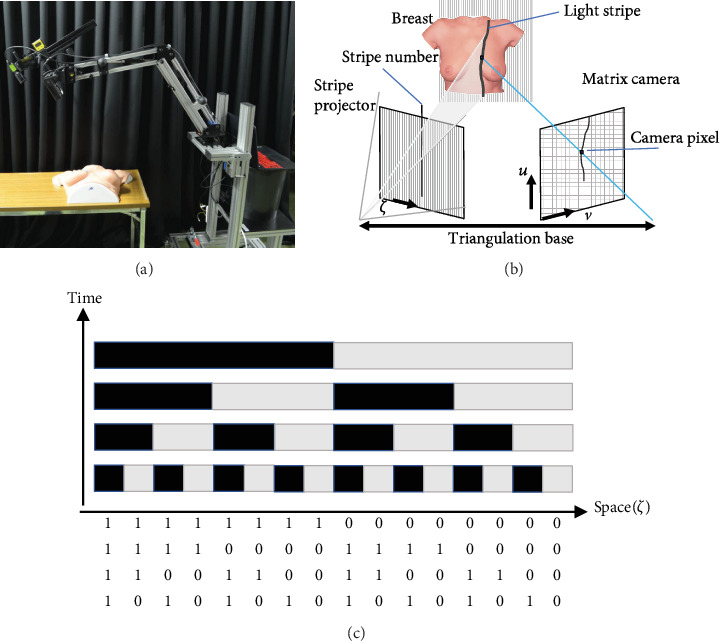
Projection mapping (PM) system. (a) The direction of projection is adjusted to match that of the maximum intensity projection image by changing the arm position and head orientation of the PM system. In this system, the relationship between the position and orientation of the projector and the camera is fixed for triangulation. (b) Triangulation. When the relationship between the position and orientation of the camera and the projector is known, the depth of the object can be calculated from the position of the projected image and the position of the camera image. (c). Structured light method. Black and white multipatterns projected onto a certain period can be encoded. The spatial position (*ζ*) can be specified by the encoded numbers.

The 3D measurement and mapping projection were installed in our prototype PM system (Figure 2).

#### 2.3.1. Hardware

Our PM system has a movable arm and tilting head with a color digital camera (CMOS Color: JAI GO-5000C; Jai, Kanagawa, Japan), 16 mm FA lens fixed-aperture f 8.0 (RICOH FL-BC1618-9M; RICOH, Tokyo, Japan), and a projector (350 lm WXGA DLP projector, ASUS P2E; ASUSTeK Computer, Taipei, Taiwan) for 3D measurement and projection. The camera and projector are fixed and calibrated.

#### 2.3.2. Processing

3D reconstructions of the breast surface were generated using the structured light method. The structured light is a process of projecting a known pattern (often using grids or horizontal bars) onto an object. The depth and surface information of the object can be calculated by observing and analyzing the deformation of the grids or horizontal bars projected onto the surface. In our system, a gray-scale, striped binary code pattern was adopted as a structured illumination technique, because it is robust to the color and subsurface scattering of the patients' skin. In addition, it is highly resistant to noise disturbance. The projector of the PM system projects multipatterns of light and dark stripes with varying widths on the area of interest: breast skin in our study. We analyzed the recorded stripe patterns, which reflected the angle and position of the camera relative to the projector. The geometrical depth was measured by triangulation and the least squares method, because triangulation can be used for a pattern of either vertical or horizontal stripes, and the combination of triangulation and least square modelling can provide stable information about the depth of any object.

#### 2.3.3. Matching and Projection

**Figure 3:**
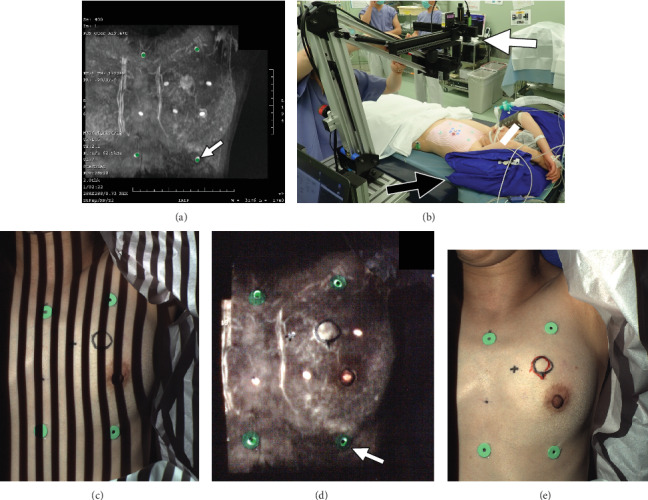
Projection mapping (PM; Case 6). (a) Maximum intensity projection (MIP) image generated from supine MRI for the PM procedure. The rigid markers on the MIP image are highlighted in green to coregister the skin and MRI markers (arrow). (b) PM procedure in the operating room. With the patient under general anesthesia, the arms are raised as with MRI scans in a supine position. Vac-Lok™ cushions (black arrow) are set under the patient. The projector of the PM system (white arrow) is set perpendicular to the floor for tumors located in the inner breast regions. (c) The depth of the breast surface is measured by observing and analyzing the deformation of the striped binary code pattern projected. (d) The MIP image projected onto the breast surface. The projection was considered successful when the MIP image projected onto the breast surface and the breast skin's rigid markers coincided (white arrow). The black outline represents the location of the cancer drawn by the conventional method prior to the PM procedure. (e) The location of a cancer drawn by the conventional method and PM method. The contour of cancer identified by the PM system is shown in red, which almost matches that by the conventional method shown in black. The positional discrepancy between the 2 methods was 4 mm.

Four or five green markers were put on the skin as rigid skin markers; the positions of which were identical to those of the Adalat® capsules used in subsequent supine MRI scanning. These green markers were used because of their easy identification on the skin. MRI scans and the breast surface were coregistered using skin and MRI markers in 3D physical coordinates. On the other hand, the bright dots of the Adalat® capsules in the MIP images being prepared to be projected were highlighted in green with a marking tool in our PM system (Figure 3(a)).

### 2.4. Conventional Method and PM Procedure in the OR

#### 2.4.1. Positioning

With the patient under general anesthesia, both arms were raised as when performing an MRI scan in the supine position (Figure 3(b)). Vac-Lok™ cushions were set under the patient's arms and back as well. The operating table was tilted to 15° for cases of cancer in the outer breast areas and set horizontally for cases of cancer in the inner breast areas.

#### 2.4.2. Definition of Cancer Location by Conventional Methods

The location of each cancer was drawn in black on the breast surface in the conventional method using palpation and US while referring the prone MRI prior to the PM procedure.

#### 2.4.3. Rigid Skin Markers

Green rigid skin markers were put on the points, which had been drawn immediately after MRI with the patient in a supine position to match the rigid markers on MIP images. Thereby, the locations of the green rigid skin markers were the same as those using the Adalat® markers.

#### 2.4.4. PM Procedure

The projector of the PM system was held vertically to the floor for tumors located in the inner breast regions. On the other hand, it was held perpendicular to the chest wall for tumors located in the outer breast regions. The angle of the MIP image was the same as that of the projector. The PM system projected patterns of light and dark stripes with varying widths on the breast skin to analyze the surface topography (Figure 3(c)). The MIP image was projected onto the breast surface using the software “Projection Mapping” in the PM system while the patients' breath was held under artificial respiration for 6 s, and the OR lights turned off. The projection was considered successful when the MIP image projected onto the breast surface and the skin's rigid markers coincided (Figure 3(d)). The contour of each invasive breast cancer identified by the PM system was drawn in red on the breast surface (Figure 3(e)). The PM method required about 15 min.

#### 2.4.5. Indigo Carmine Injection

A surgical line was drawn on the breast surface with a 10–15 mm margin from the contour of each cancer determined by the conventional method as usual. Indigo carmine was injected towards the breast tissue to reach just above the greater pectoral muscle from the surgical line at intervals of 10–15 mm at the same angle as that of the projector of the PM system, while keeping the patient's arms raised. After that, the Vac-Lok was removed and both arms were spread sideways as the subsequent operative position. The cylindrical resection guided by the breast tissue stained with indigo carmine was performed.

### 2.5. Feasibility of the PM System and Statistical Methods

We evaluated the feasibility of our PM system in the OR by the tumor size and location [7]. First, we compare the maximum diameters of tumors between the PM method, the conventional method, and pathology. The Kruskal-Wallis test was used for comparison. *P* < 0.05 was considered to indicate statistically significant. Next, the locations of invasive breast cancers detected by the PM system were compared with those identified by the conventional method. Discrepancies in cancer location were defined as the maximum distance between the edge of the invasive cancer drawn by these two methods. In this study, the discrepancy > 5 mm was considered inadequate: the surgical margin from cancerous cells could be less than 5 mm and the cells were close to spread to the tissues outside the surgical margin when a surgical line for cylindrical resection of the cancer was drawn with a 10 mm margin from the contour of cancer.

## 3. Results

Two of the 10 patients did not complete the PM procedure because of our errors: Vac-Lok™ cushions were put below one patient's back inappropriately and the projector of the PM system was tilted at an invalid angle in the other.

The maximum diameters of invasive breast cancers ranged from 9 to 24 mm on the conventional method (16.8 ± 5.1 mm), from 8 to 24 mm on PM system (14.9 ± 4.9 mm), and from 6 to 25 mm on pathology investigations (15.3 ± 7.2 mm). There was no statistically significant difference in the maximum diameter between MIP, US, and pathology (*P* = 0.84). The discrepancy of the cancer location between the PM system and the conventional method was measured at 3–8 mm (4.5 ± 1.6 mm). This discrepancy was judged acceptable (i.e., <5 mm) in seven of the eight patients. Table 1 summarizes the surgical findings and maximum diameters of invasive cancer shown by the PM method, conventional methods, and pathology.

**Figure 4:**
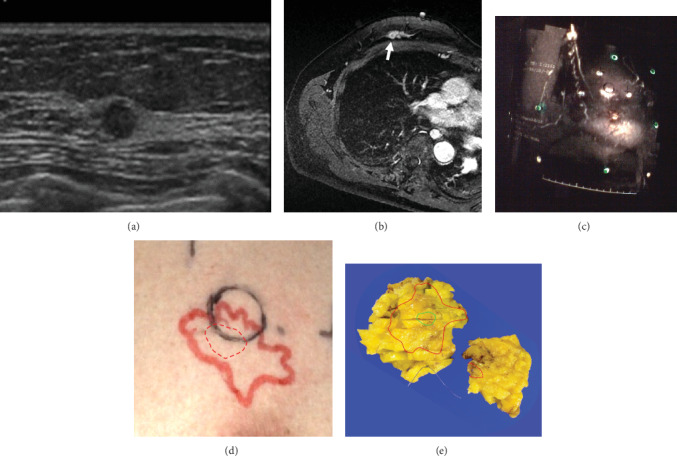
59-year old woman with breast cancer in the outer portion (Case 10). Comparison between the conventional method, PM (projection mapping) method, and pathological findings (Case 10). (a) Sonography (US) shows invasive cancer as a round-shaped low echoic mass but not ductal carcinoma in situ (DCIS). (b) Supine MRI using home-made tilting table shows the entire spread of breast cancer as nonmass enhancement including the invasive part showing a small nodule with rim enhancement (arrow). (c) The nonmass enhancement on the MIP image generated from supine MRI is projected onto the breast skin. The black circle is the location of the invasive cancer drawn using US. (d) The PM method is able to identify tumor spread including DCIS (red). The dotted red circle represents the location of the invasive cancer in the nonmass enhancement, which is not identical to that by US (black) perfectly. (e) The extent of cancer by the PM method is consistent with that of pathological findings (green: invasive cancer; red: DCIS).

Figure 4 shows the case where the discrepancy of the location of the invasive cancer between the PM system and the conventional method was largest (i.e., 8 mm), and the PM method visualized clearly the surrounding DCIS invisible by US (Case 10).

## 4. Discussion

This study demonstrated that the maximum diameters of invasive breast cancers did not differ significantly between the PM system and conventional method or pathology. The mean discrepancy in cancer location identified using our PM system and the conventional method was 4.5 mm, i.e., less than 5 mm, in almost all patients. This discrepancy is considered clinically relevant, as the surgical margin should be 10–15 mm from the edge of the cancer. Thus, the PM system may be acceptable for supporting breast-conserving surgery by demonstrating the tumor size and location on the breast surface accurately. To our knowledge, this is the first report to describe the technique projecting breast MRI images onto the breast skin using a projector.

MRI is the best imaging modality for evaluating breast cancer location and spread. The MIP image visualizes the entire image of each cancer, including its anatomical relationship with the nipple and any associated DCIS, because it can display the cancer from any angle with a wide range of view. However, breast MRI is routinely done in a prone position, unlike the supine position required on the operating table. Some reports have showed that preoperative MRI scans in the prone position are not useful for breast-conserving surgery because they can increase the rate of mastectomy and additionally fail to reduce the reoperation rates caused by positive margins [10, 11]. Other studies have performed supine breast MRI before surgery [12–15]. The present PM system may be more feasible for breast-conserving therapy, because it was used in OR immediately before the surgery and the structure light method used here corrected the MIP images according to the breast shape. Real-time virtual US, which synchronizes MRI and US on one imaging monitor, has emerged as a new technique [13, 14]. Nevertheless, US has difficulty in identifying comprehensive images of breast cancer and is still operator-dependent. CT is performed in the same supine position as surgery, but the contrast between cancer and breast tissues is lower than MRI.

We newly developed a prototype PM system to project MIP images from supine MRI scans on the patient's breast skin when drawing surgical lines for breast-conserving surgery in the OR. A structured light method was installed in this system for 3D surface measurements and correcting distortions. This method is also used in a patient positioning system for radiation therapy [16, 17]. The projector in this system was used not only for projecting the MRI images but also for the structured light projection method.

Supine MRI was performed with the aim of reproducing the same shape of the breast between the MRI scans and on the operating table [12]. We requested patients to relax and to breathe quietly during MRI scans and used Vac-Lok™ shape-memory cushions used for radiation therapy, to fix the arms both during the MRI scans and on the operating table. In this way, we successfully projected the MIP images onto patients' breast surfaces using our PM system in the OR, and consequently, the maximum diameters and locations of invasive cancer identified by the system were consistent with those identified by conventional methods and pathology. Nevertheless, we think that it is still inappropriate to omit a prone MRI scan because it shows breast cancer more clearly than supine MRI. Actually, we referred prone MRI findings when determining the spread of cancer by supine MRI.

In one patient, the discrepancy in cancer location between our PM system and the conventional method was 8 mm as presented in this study: the tumor-free margin distance may be only 2 mm. Behm et al. [3] report that the distance of 2 mm should be adopted as an adequate margin for excision for invasive breast cancer. Further studies will be necessary to minimize such discrepancies in patients with tumors in outer regions or in cases of soft breasts changing their shape.

There were a couple of limitations to this preliminary study. First, the sample size was small. In addition, there were few patients with cancers in the outer breast regions, although the outer-upper region is mostly involved in the general population. The outer region can change its shape markedly, which required us to perform supine MRI with a tilting table and to tilt the operating table carefully. Second, breast-conserving surgery was performed by conventional methods in this study. Therefore, we could not evaluate whether the surgical margin was positive or negative in surgery performed at the resection line determined by the PM system. Indeed, five of the 10 patients in this study had DCIS. MRI is well suitable for detecting the extent of DCIS associated with invasive breast tumors. This MRI-based PM system should be feasible for breast-conserving surgery for small invasive cancers with DCIS components or pure DCIS. Third, the location of the cancer projected onto the breast skin by this PM system is useful for drawing the surgical line for the cylindrical resection established in some countries, but there is no depth information of the cancer required for other localization techniques, such as hook-wire-guided localization.

## 5. Conclusions

Our MRI-based PM system used in the OR could identify the size and location of breast cancer accurately in this preliminary clinical study. This PM system may eliminate the labor of repositioning MRI images in the “surgeons' brains” in the OR.

## Figures and Tables

**Table 1:** Assessment of invasive breast cancer by projection mapping, conventional method, and pathology.

Patient		Breast cancer					PM procedure			Breast conserving surgery		Maximum diameter of invasive part(mm)			
**No.**	Age (y/0)	Side	Inner/outer	Histology and grade	TN classification	Additional lesions	Slope	Incidence angle (degree)	Rigid marker	Margin (mm)	Margin involvement	Pathology	Conventional method	PM method	Discrepancy^∗∗^
**1**	66	Right	Inner	IDC gr2	T1cN0	DCIS	No	0	Match	10	Negative	15	12	14	3
**2**	62	Right	Inner	IDC gr2	T2N1a	—	No	0	Match	10	Negative	25	24	20	5
**3**	60	Left	Inner	IDC gr2	T1cN0	DCIS < 5 mm	No	0	Match	10	Negative	18	15	15	3
**4**	53	Left	Outer	IDC gr1	T1bN0	DCIS	Yes	5	Match	15	Negative	8	16	12	5
**5**	60	Left	Inner	IDC gr1	T1bN0	—	No	0	Mismatch^∗^	15	Negative	8	—	NA	NA
**6**	36	Left	Inner	IDC gr3	T1cN0	DCIS	No	0	Match	15	DCIS	18	20	16	4
**7**	70	Left	Outer	IDC gr2	T2N0	DCIS < 5 mm	Yes	5	Mismatch^∗^	NA	Negative	32	35	NA	NA
**8**	45	Left	Inner	IDC gr3	T2N0	—	No	0	Match	15	Negative	25	24	24	4
**9**	45	Left	Inner	IDC gr1	T1bN0	—	No	0	Match	15	Negative	7	9	8	4
**10**	59	Right	Outer	IDC gr2	T1bN0	DCIS	Yes	20	Match	10	DCIS	6	14	10	8

PM: projection mapping; US: ultrasound; DCIS: ductal carcinoma in situ. ^∗^Patients who could not compete the PM procedure because of human errors. ^∗∗^Discrepancies in cancer location were defined as the maximum distance between the edge of the invasive cancer drawn by these two methods.

## Data Availability

The data used to support the findings of this study are available from the corresponding author upon request.

## References

[1] Litière S., Werutsky G., Fentiman I. S. (2012). Breast conserving therapy versus mastectomy for stage I-II breast cancer: 20 year follow-up of the EORTC 10801 phase 3 randomised trial. *The Lancet Oncology*.

[2] Meric F., Mirza N. Q., Vlastos G. (2003). Positive surgical margins and ipsilateral breast tumor recurrence predict disease-specific survival after breast-conserving therapy. *Cancer*.

[3] Behm E. C., Beckmann K. R., Dahlstrom J. E. (2013). Surgical margins and risk of locoregional recurrence in invasive breast cancer: an analysis of 10-year data from the Breast Cancer Treatment Quality Assurance Project. *Breast*.

[4] Leithner D., Wengert G. J., Helbich T. H. (2018). Clinical role of breast MRI now and going forward. *Clinical Radiology*.

[5] Sardanelli F., Boetes C., Borisch B. (2010). Magnetic resonance imaging of the breast: recommendations from the EUSOMA working group. *European Journal of Cancer*.

[6] Satake H., Ishigaki S., Kitano M., Naganawa S. (2016). Prediction of prone-to-supine tumor displacement in the breast using patient position change: investigation with prone MRI and supine CT. *Breast Cancer*.

[7] Joukainen S., Masarwah A., Könönen M. (2019). Feasibility of mapping breast cancer with supine breast MRI in patients scheduled for oncoplastic surgery. *European Radiology*.

[8] Van der Jeught S., Dirckx J. J. (2016). Real-time structured light profilometry: a review. *Optics and Lasers in Engineering*.

[9] Rusinkiewicz S., Hall-Holt O., Levoy M. (2002). Real-time 3D model acquisition. *ACM Transactions on Graphics*.

[10] Turnbull L., Brown S., Harvey I. (2010). Comparative effectiveness of MRI in breast cancer (COMICE) trial: a randomised controlled trial. *Lancet*.

[11] Houssami N., Turner R. M., Morrow M. (2017). Meta-analysis of pre-operative magnetic resonance imaging (MRI) and surgical treatment for breast cancer. *Breast Cancer Research and Treatment*.

[12] Sakakibara M., Yokomizo J., Shiina N. (2014). MRI-guided quadrantectomy in patients with ductal carcinoma in situ detected preoperatively by mammographic calcifications. *Journal of the American College of Surgeons*.

[13] Nakano S., Kousaka J., Fujii K. (2012). Impact of real-time virtual sonography, a coordinated sonography and MRI system that uses an image fusion technique, on the sonographic evaluation of MRI-detected lesions of the breast in second-look sonography. *Breast Cancer Research and Treatment*.

[14] Uematsu T., Takahashi K., Nishimura S. (2016). Real-time virtual sonography examination and biopsy for suspicious breast lesions identified on MRI alone. *European Radiology*.

[15] Pallone M. J., Poplack S. P., Avutu H. B., Paulsen K. D., Barth R. J. (2014). Supine breast MRI and 3D optical scanning: a novel approach to improve tumor localization for breast conserving surgery. *Annals of Surgical Oncology*.

[16] Peng J. L., Kahler D., Li J. G. (2010). Characterization of a real-time surface image-guided stereotactic positioning system. *Medical Physics*.

[17] Lindl B. L., Müller R. G., Lang S., Herraiz Lablanca M. D., Klöck S. (2013). TOPOS: a new topometric patient positioning and tracking system for radiation therapy based on structured white light. *Medical Physics*.

